# The epidemiological characteristics of neurogenic limb deformity disorder in China: a national-based study from Qin Sihe orthopedic center

**DOI:** 10.1186/s12889-023-15470-9

**Published:** 2023-03-27

**Authors:** Kai-bing Liu, Jack Guan, Jian-cheng Zang, Qi-kai Hua, Si-he Qin

**Affiliations:** 1 Department of Bone and Joint Surgery, The First Affiliated Hospital of Guangxi Medical University, Nanning, China; 2 Bay Area Foot and Ankle Medical Clinic, San Jose, USA; 3 Department of Orthopaedics, Rehabilitation hospital, National Research Center For Rehabilitation, Beijing, China; 4 Key Laboratory of Intelligent Control and Rehabilitation Technology, Ministry of Civil Affairs, Beijing, China

**Keywords:** Neurogenic limb deformity disorder, Orthopedics, China, Epidemiology

## Abstract

**Background:**

Neurogenic limb deformity disorder (NLDD) refers to limb deformity disorders caused by various neurogenic disorders. However, there are no studies to systematically summarize and analyze these diseases in China, and we first proposed the concept of NLDD. We describe the epidemiological characteristics of NLDD in China based on the largest case database of limb orthopedics in China.

**Methods:**

This study analyzed parameters from the Qin Sihe Orthopedic Surgery Case Data (QSHOSCD). The database is based on the Rehabilitation Hospital affiliated to National Research Center for Rehabilitation, which has collected nearly 37,000 patients to date and includes a wide variety of limb deformities. The types of diseases are summarized and classified for all patients studied. Statistical analysis was based on the type of etiology, age, regional distribution, and historical surgical volume. Partial outcomes were statistically analyzed separately by common diseases (polio and cerebral palsy) and rare diseases (37 other diseases).

**Results:**

From 1979 to 2019, 30,194 patients with NLDD were treated surgically for 39 neurogenic disorders. The male to female ratio was 1.48:1, the mean age was 19.65 years, and most patients (82.38%) were aged between 6 and 30 years. Patients included from 32 provinces and cities across China, mainly concentrated in populous central provinces and Heilongjiang Province. The peak of annual surgical procedures was from 1988 to 1994, and the number of annual surgical procedures for common diseases gradually decreased from 1994 onwards, but the trending is opposite for rare diseases.

**Conclusions:**

This study is the first to demonstrate the disease types, population characteristics and incidence trends of NLDD in China. It suggests that the prevention and treatment of NLDD should focus on the adolescent population and enhance the treatment of neurogenic diseases that cause limb deformities. The growth and adaption of the Ilizarov technique and its practice in Chinese orthopedic benefits the treatment of neurogenic limb deformity disorders.

## Background

Limb deformities define as a group of common dysmorphic disorders, mainly manifested by abnormal limb appearance and functional impairment. Neurogenic limb deformity disorder (NLDD) refers to limb deformity disorders caused by various neurogenic diseases, and we propose this concept for the first time. Currently there are relatively few studies on NLDD [1], these studies are mainly summary analyses of individual disorders, such as polio [2, 3] and cerebral palsy [4–6]. In China, no studies have reported on the epidemiological characteristics of NLDD.

According to the 2010 National Disability Census statistics released by the China Disabled Persons’ Federation [7], China has 24.72 million people with limb disabilities. Therefore, revealing the Epidemiological characteristics of NLDD in China for the prevention and treatment of such diseases has great potential impact.

Data from the Qin Sihe Orthopedic Surgery Case Data (QSHOSCD) was used to analyze the disease type, sex, age, regional distribution and number of surgeries from 1979 to 2019 for neurogenic limb deformity disorder. This study aims to summarize the distribution and characteristics of NLDD in China, as well as the epidemic trends.

## Methods

### Diagnosis standard

Neurogenic limb dysmorphic disorder (NLDD) refers to limb dysmorphic disorder caused by various neurogenic diseases. Its diagnosis should meet the following conditions: (1) A clear history of neurogenic disease. (2) Impaired or abnormal neurological function. (3) Deformity and dysfunction of limbs (congenital and other causes of deformity must be excluded).

### Study design

This retrospective study used data from the QSHOSCD. The information collected in the QSHOSCD was based on informed consent from patients, and patient privacy was strictly protected in accordance with relevant guidelines and regulations.

### Participants/study subjects

The QSHOSCD was established by Dr. Qin Sihe’s team at the Rehabilitation Hospital affiliated to National Research Center for Rehabilitation. The database has collected nearly 37,000 cases to date, including a wide variety of limb deformities. Each patient was counted as one case regardless of the number of surgical procedures during the same hospitalization. Multiple inpatient surgeries in different hospitalizations were counted as separate cases. Statistics on etiological species are summarized and classified based on etiology to determine the types of diseases that lead to limb deformity disability, and neurogenic diseases are screened according to the current clinical disciplinary subgenus. In total, we collected 30,194 cases of NLDD from 1979 to 2019 in the QSHOSCD.

### Variables, outcome measures

The patients’ disease composition, gender, age, regional distribution, and number of surgeries over the years were statistically analyzed. Considering that the number of polio (79%) and cerebral palsy (16%) surgery cases accounted for a high proportion of the total cases, the common diseases (polio and cerebral palsy) were separated from the rare diseases (37 other causes) in the analysis of some factors.

### Statistical analysis

All variables analyzed using frequencies, and percentages. A chi-square test was used to compare the differences in sex ratios between common and rare diseases. P < 0.05.

was designated as statistically significant. Statistical analyses were performed using SPSS version 20.0 (IBM, Armonk, NY, USA).

## Results

### Disease composition of NLDD

The 30,194 patients covered 39 different neurological disorders, spanning different disciplines such as internal medicine, general surgery, gynecology, and pediatrics. The four most common disease types were polio (23,757 cases), cerebral palsy (4767 cases), spina bifida/spinal cord embolism (975 cases), and motor neuron disease (250 cases) (Table 1). Polio (79%) and cerebral palsy (16%) account for a large proportion of NLDD (Table 1).

**Table 1:** Composition of 39 neurogenic disorders causing limb deformity disability

Diseases	Cases (n)	Proportion (%)	Diseases	Cases (n)	Proportion (%)
Polio	23,757	78.68	Spasticity of spinal origin	2	0.01
Cerebral palsy	4767	15.79	Cerebral tuberculosis	2	0.01
Spina bifida/ TCS	975	3.23	Thoracic spinal cord compression	2	0.01
Motor neuron disease	250	0.83	Epileptic sequelae	1	0.00
Guillain-Barre syndrome	84	0.28	MPNST	1	0.00
Cerebritis	74	0.25	Peripheral sensory neuropathy	1	0.00
Brain trauma	64	0.21	Spinal arachnoiditis	1	0.00
Meningitis	36	0.12	Tuberculous meningitis	1	0.00
Lateral sclerosis	33	0.11	Intracranial cyst	1	0.00
Hydrocephalus	22	0.07	Intracranial germ cell tumor	1	0.00
Obstetrical palsy	21	0.07	Spongiform degeneration	1	0.00
Acute myelitis	20	0.07	Cerebral vasculitis	1	0.00
Hereditary spastic paraplegia	20	0.07	Choriomeningitis	1	0.00
Epidemic encephalitis B	11	0.04	Cerebrovascular malformations	1	0.00
Hand, foot, and mouth disease	11	0.04	Cerebral thrombosis	1	0.00
Spinal muscular atrophy	9	0.03	Cerebral arachnoiditis	1	0.00
Spinal cord injury	7	0.02	Parkinson’s disease	1	0.00
Stroke	6	0.02	Peripheral nerve palsy	1	0.00
Wilson’s disease	3	0.01	Subarachnoid haemorrhage	1	0.00
Transverse myelitis	2	0.01			0.00

Abbreviations: *TCS* tethered cord syndrome, *MPNST* malignant peripheral nerve sheath tumor

### Population distribution

#### Sex distribution

In total, 18,028 male (59.71%) and 12,166 female (40.29%) were included in this study, with a male-to-female ratio of 1.48:1. The sex ratio was significantly different (χ^2^ = 38.65, p < 0.001) for common (1.51) and rare (1.10) diseases, as shown in Table 2.

**Table 2:** Gender distribution of patients

Diseases	Gender		Sex ratio, M/F	χ^2^	p value
	Male (%)	Female (%)			
Common diseases	17,152(60.13)	11,372(39.87)	1.51	38.65	<0.001
Rare diseases	876(52.46)	794(47.54)	1.10		
Total	18,028(59.71)	12,166(40.29)	1.48		

*Abbreviations*: *M/F* Males/Females

#### Age distribution

**Fig. 1:**
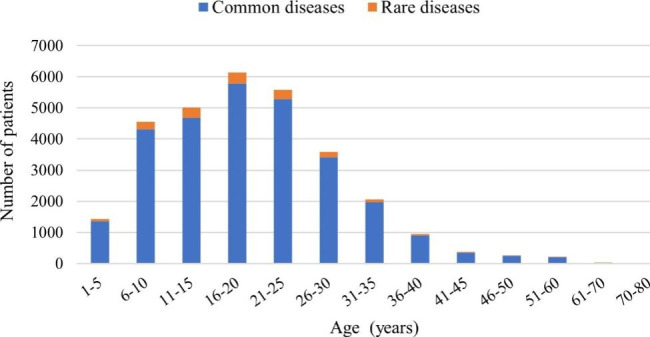
Age distribution of patients

The mean age of NLDD patients was 19.65 years. However, the age span was large, with the youngest age being 1 year and the oldest age being 73 years (Table 3). The age distribution of NLDD patients was characterized by a high prevalence starting in childhood and peaking around the age of 20 years. 82.39% of the patients were between the ages of 6–30 years. The number of patients receiving surgery after the age of 30 years showed a gradual decline, reaching low levels after 60 years (Table 3). The age distribution was similar for the common and rare diseases (Fig. 1)

**Table 3:** Age distribution of patients

Age group	Common diseases		Rare diseases		Total cases (n)	Total proportion (%)
	Cases (n)	Proportion (%)	Cases (n)	Proportion (%)		
1–5	1363	4.78	63	3.77	1426	4.72
6–10	4310	15.11	244	14.61	4554	15.08
11–15	4683	16.42	333	19.94	5016	16.61
16–20	5780	20.26	362	21.68	6142	20.34
21–25	5283	18.52	294	17.60	5577	18.47
26–30	3409	11.95	178	10.66	3587	11.88
31–35	1970	6.91	87	5.21	2057	6.81
36–40	902	3.16	49	2.93	951	3.15
41–45	343	1.20	33	1.98	376	1.25
46–50	245	0.86	15	0.90	260	0.86
51–60	209	0.73	8	0.48	217	0.72
61–70	25	0.09	3	0.18	28	0.09
70–80	2	0.01	1	0.06	3	0.01

In all cases, the oldest age was 73 years, the youngest age was 1 year, and the average age was 19.65 years

#### Regional distribution

**Fig. 2:**
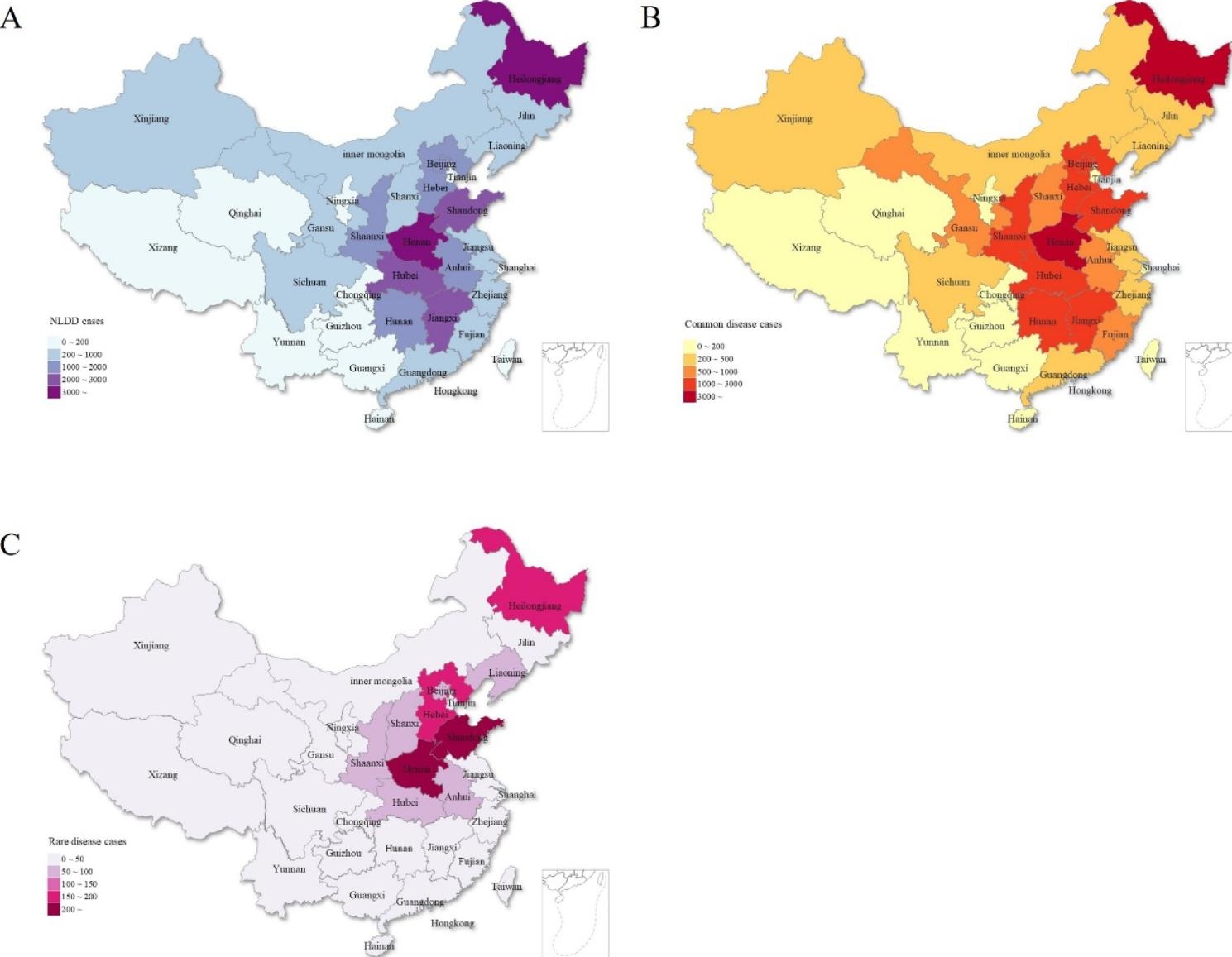
Map of the regional distribution of patients with NLDD (A), common diseases (B) and rare diseases (C) between 1979 and 2019

The patients originated from 33 provinces, municipalities, and autonomous regions of China (Fig. 2A). The Heilongjiang province had the highest number of patients, with 6,302 cases, accounting for 20.87% of NLDD cases. We found that patients with NLDD were mainly concentrated in central China, while there were fewer patients in the eastern coastal areas (Fig. 2A). There were even fewer patients in the remote mountainous areas of the west, with many provinces having fewer than 200 cases (Fig. 2A). In addition, the number of common disease cases was highest in Heilongjiang province, and the number of rare disease cases was highest in Henan province, and the distribution in other regions, both similar. (Figure 2B C).

### Annual surgery volume

**Fig. 3:**
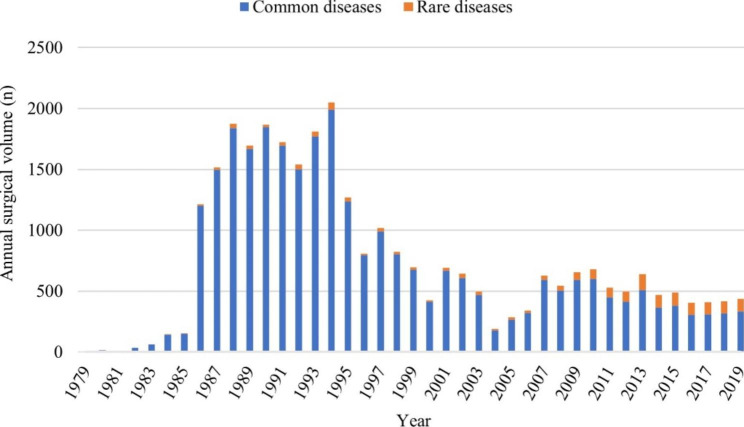
The volume of surgeries in the calendar year from 1979 to 2019

**Fig. 4:**
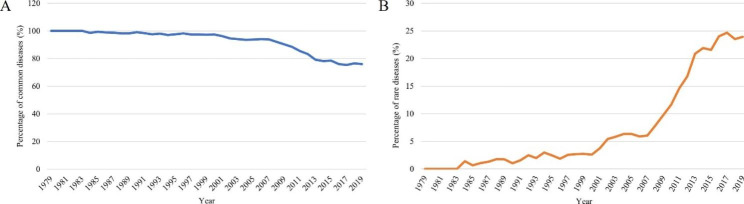
Percentage of common diseases (A) and rare diseases (B) in the calendar years 1979 to 2019

Dr. Qin Sihe did the first case of NLDD in 1979, and for more than 40 years, we have been working on the orthopedic treatment and rehabilitation of patients with NLDD. The annual surgical volume began to increase slowly in 1979 and increased rapidly after 1986, peaking at an average of more than 1,500 cases per year from 1988 to 1994. After 1995, the annual number of surgical began to decrease gradually and tended to be relatively stable after 2007 (Fig. 3). In contrast, the annual surgical volume of rare diseases gradually increased after 2007 and remained at a steady level (Fig. 3). In the 21st century, the proportion of surgical volume of common diseases gradually decreased and that of rare diseases gradually increased (Fig. 4A and B). The proportion of rare diseases increased rapidly after 2007 and remained at a level of more than 20% after 2013 (Fig. 4B).

## Discussion

Limb deformities due to individual diseases such as poliomyelitis and cerebral palsy have been studied by previous authors [2–4, 6], few have organized these diseases into a category for summary and study [1]. In this study, the characteristics of 30,194 NLDD patients treated surgically between 1979 and 2019 based on the QSHOSCD were statistically analyzed. We characterize Chinese NLDD patients by a male predominance, a high prevalence in the adolescent population, and a wide geographical distribution.

This study revealed a variety of causes spanning different disciplines such as internal medicine, orthopedic, gynecology, and pediatrics and covering 39 different neurological diseases. The etiology with the highest number of surgical cases was polio (poliomyelitis). Polio is an infectious disease caused by the poliovirus [8], with severe manifestations leading to temporary or permanent paralysis [9]. Polio patients usually show asymmetric limb and muscle involvement, muscle atrophy, and bone dysplasia, resulting in characteristic musculoskeletal deformities. Musculoskeletal deformities can manifest as leg length discrepancies, joint misalignment, spinal deformities and joint contractures [10]. In the 1980s, polio was endemic in 125 countries, and many children survived with limb paralysis, subsequently developing severe lifelong disabilities mainly affecting their legs [11]. Polio has traditionally been an endemic disease in China and is considered a significant cause of severe disability and death. Between 1965 and 1977, 4500–29000 cases per year were reported [12]. Thus, polio has the highest number of cases in NLDD.

There were more males than females in all patients and there was a significant difference in the sex ratio in common and rare diseases (p < 0.001). We have no evidence to support that the onset of NLDD is associated with gender. Due to last century’s gender imbalance. Starting the end of 20th century, the gender ratio in China is 1.05:1 [13]. The age of patients at the time of surgery spanned a wide range, with a mean age of 19.65 years. A high prevalence was observed from childhood, with a peak around the age of 20 years. Surgical treatment for neurologic limb deformity was concentrated in adolescents. These characteristics are similar to our previous study and are related to the high motor activity and high need to participate in social activities in this age group [14], with a strong demand to restore healthy aesthetics and improve limb function. Numerous studies have shown that poliovirus affects mainly infants and children, leading to lifelong disability or death [15]. In addition, polio that develops prior to a growth spurt usually leads to progressive scoliosis and limb shortening [16].Spastic cerebral palsy is the most common form of cerebral palsy, accounting for 80% of all cases [17]. Children with spastic cerebral palsy manifest gradual contracture of the fixed leg muscles, resulting in a significant reduction in passive range of motion in hip flexion and abduction, knee flexion, and ankle dorsiflexion [18–20]. According to Pauwel’s law, untreated fixed muscle contractures may lead to changes in skeletal shape and eventually to musculoskeletal deformities as the affected child grows and develops [21]. These studies explain why the main population of NLDD was adolescents, mainly related to their rapid growth and development. There are also a few patients between the ages of 30 and 60. Some studies have shown that after a long period of stabilization (usually decades), many patients with residual damage from paralytic polio develop new disabilities [22]. There may also be progressive wasting and weakness in limbs already affected by polio, and compensatory hypertrophy may be observed in the contralateral limb [23].

Although this is a single-center study, we have a wide geographical distribution of patients from 33 provinces, municipalities, and autonomous regions in China. The largest number of NLDD patients is in Heilongjiang province (6302 cases), accounting for 20.87% of the total. This is related to the fact that the corresponding author had worked in the Heilongjiang province for 5 years. We found that NLDD patients are mainly concentrated in central China, this is consistent with the fact that China’s population distribution has historically been concentrated in Central China. The economic development of the eastern coastal region of China, with better medical treatments is such that the primary disease of NLDD is well treated, therefore are fewer patients with NLDD. A medical geography big data set analysis showed that China’s healthcare resources are spatially unbalanced, with the abundance and balance of healthcare resources generally better in the east than in the west [24]. It is difficult for NLDD patients in remote areas in the west to get good medical resources, and for us to collect this group of patients. Our next is goal is shifting our focus to that China should give more support to the western remote mountainous areas of China for NLDD prevention and treatment.

The annual surgical volume increased rapidly after 1986, peaking from 1988 to 1994. This coincided with the implementation of post-polio salvage surgery rehabilitation in China. The corresponding author was the director of both the Heilongjiang and Beijing polio sequelae correction centers, presiding over the surgical correction tasks in both provinces [14]. Therefore, we have operated on many NLDD patients during this period. In fact, universal polio vaccination in China began in 1965, and after 1978, oral polio vaccine became a routine vaccination for all children in the country[12]. WHO launched the Global Polio Eradication Initiative (GPEI) in 1988 [25], and in 1989, Chinese government leaders made a political commitment to eradicate polio in China. Notably, China reported its last indigenous case of wild poliovirus infection in September 1994 [26]. The eradication of indigenous polio in China and the annual number of NLDD surgeries also began to decrease gradually after 1995, and tended to be relatively stable after 2007. In the 1990s, Dr. Qin Sihe and others introduced Ilizarov technology from Russia [27], and the Ilizarov external fixation frame was gradually applied in the correction of various complex limb deformities, and the development of orthopedic surgery in China became better and better. We have accumulated a wealth of clinical experiences in the treatment of various limb deformities and have gradually developed the concept of natural reconstruction in orthopedics [28]. Learning from our previous experiences, we are now able to treat and service more patients with rarely seen diseases. Overall, the annual volume of NLDD procedures is still decreasing, which in a sense reflects the decreasing incidence of NLDD.

The limitations of this study as follows. First, although the number of patients included in this study was as high as 30,194, all of these patients were from QSHOSCD, and lacking sampling weights, the data set could not include the entire Chinese incidence population. Patients from more centers should be included when assessing the prevalence of NLDD in China. Second, the included patients spanned over 40 years from 1979 to 2019, making it difficult to follow up on postoperative complications and long-term function. Third, this study involves a wide range of diseases, and there is a possibility of misdiagnosis leading to inaccurate results, especially for rare diseases with complex etiologies.

## Conclusion

Our study is the first study to summarize and analyze the epidemiological characteristics of NLDD in China, with a high prevalence in the adolescent population, a regional distribution concentrated in the central and northeastern regions, and a current low level of incidence. It suggests that the prevention and treatment of NLDD should focus on the adolescent population and enhance the treatment of neurogenic diseases that cause limb deformities. Through ilizarov techniques will be bring a better future for all the patients whom suffer neurologic limb deformities.

## Data Availability

The datasets used during the current study are available from the corresponding author on reasonable request.

## Abbreviations

NLDD: Neurogenic limb deformity disorder
QSHOSCD: Qin Sihe Orthopedic Surgery Case Data
TCS: tethered cord syndrome
MPNST: malignant peripheral nerve sheath tumor
M/F: Males/Females
